# Supplementary material for: Two Ways of Targeting a CD19 Positive Relapse of Acute Lymphoblastic Leukaemia after Anti-CD19 CAR-T Cells
Source: Biomedicines. 2023 Jan 25;11(2):345. doi: 10.3390/biomedicines11020345 (PMC9953531; doi:10.3390/biomedicines11020345)
Supplement: Supplementary file 1 [file biomedicines-11-00345-s001.zip › biomedicines-2177184-supplementary.pdf]

SUPPLEMENTARY MATERIAL:

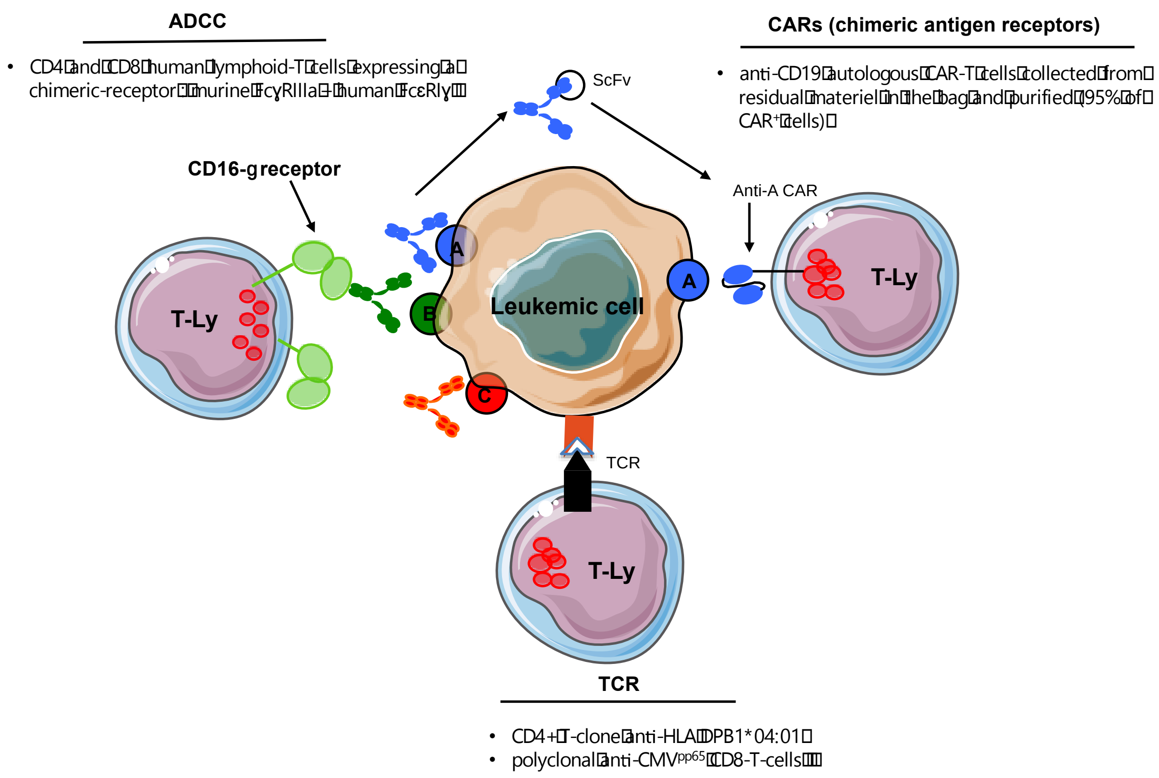

Figure S1: T-cell effectors used in the cytotoxicity assay. To compare the lysis sensitivity of each ALL, some T-lymphoid effectors were used, capable of inducing targeted cell death via different mechanisms: purified autologous anti-CD19 CAR-T cells inducing lysis through CD19 recognition by the CAR; CD4+ T-clone anti-HLA DPB1*04:01 and polyclonal anti-CMVpp65 CD8-T-cells inducing cell lysis through the TCR; and CD4 and CD8 human lymphoid T-cells transduced by a lentiviral vector, expressing a chimeric-receptor containing the murine CD16 receptor inducing cell lysis when combined with murine antibodies through the ADCC mechanism.

BLANK	CD244	DLL1	HLA-DR
Hamster IgG ctrl	CD245	DLL4	Ig light chain lambda
CCR10	CD25	DR3	IgD
CD278	CD252	EGFR	IL-28RA
IFN-gRb	CD261	GITR	Integrin b5
Mouse IgG1 ctrl	CD262	GPR19	KLRG1
CD46	CD263	GPR56	LOX-1s
CD70	CD266	HLA-Es	MICA-MICB
CD1a	CD268	HVEM	MSC W3D5
CD2	CD27	Ig light chain kappa	Notch-2
b2m	CD271	IgM	TACSTD2
B7-H4	CD275	IL-21R	TIGIT
Cadherin 11	CD276	Integrin a9b1	Mouse IgG2b ctrl
CD10	CD277	Jagged 2	C3AR
CD100	CD279	Ksp37	CCX-CKR
CD103	CD28	LAP	CD11c
CD105	CD29	LY6G6D	CD129
CD106	CD290	MERTK	CD158
CD107a	CD298	MSC W7C6	CD181
CD107b	CD3	MSC NPC W4A5	CD193
CD109	CD30	MSCA	CD196
CD111	CD300c	MUC-13	CD1d
CD112	CD309	NKp80	CD20
CD114	CD31	Notch-1	CD22
CD116	CD314	Notch-3	CD220
CD117	CD317	Notch-4	CD235ab
CD119	CD324	NPC	CD258
CD11a	CD325	NTB-A	CD274
CD11b	CD328	PSMA	CD319
CD122	CD33	ROR1	CD32
CD123	CD334	Siglec-10	CD326
CD126	CD335	Siglec-7	CD338
CD127	CD336	Siglec-8	CD368
CD13	CD337	Siglec-9	CD45RA
CD131	CD34	SSEA-5	CD45RB
CD134	CD340	SUSD2	CD49e
CD135	CD344	TCRab	CD52
CD137	CD35	TCR gd	CD66ace
CD137L	CD354	Tim-4	CD85h
CD138	CD360	TLT-2	CD85j
CD14	CD365	TM4SF20	CD86
CD140a	CD366	TRA-2-49	CD92
CD140b	CD367	TRA-2-54	CXCR7
CD141	CD36L1	TSLPR	Delta opioid R
CD142	CD38	VEGFR3	DRD1
CD143	CD39	Mouse IgG2a ctrl	EphA2
CD146	CD4	APCDD1	FceRIa
CD148	CD40	BTLA	GARP
CD15	CD41	CCR8	IL-15Ra
CD150	CD42b	CCRL2	LT-bR
CD151	CD43	CD102	MRGX2
CD154	CD44	CD104	TMEM8A
CD156c	CD45	CD124	CD254
CD158e1	CD47	CD130	CD318
CD16	CD48	CD144	Mouse IgG3 ctrl
CD161	CD49a	CD152	CD255
CD162	CD49b	CD155	SSEA-4
CD163	CD49c	CD158b	Mouse IgM ctrl
CD164	CD49d	CD184	Sialyl Lewis X
CD165	CD5	CD186	TRA-1-80
CD166	CD50	CD192	CD160
CD169	CD54	CD197	CD57
CD170	CD55	CD199	CD66b
CD172ab	CD56	CD209	TRA-1-60R
CD172g	CD58	CD217	Rat IgG1 ctrl
CD178	CD6	CD230	CD115
CD179a	CD61	CD24	CD201
CD179b	CD62E	CD243	Rat IgG2a
CD18	CD62L	CD26	CD120b
CD180	CD62P	CD269	CD210
CD182	CD63	CD282	CD267s
CD183	CD64	CD284	CD294
CD185	CD69	CD301	CD49f
CD19	CD73	CD303	CD85a
CD191	CD74	CD304	CD85d
CD194	CD79b	CD307	IgG Fc
CD1b	CD8	CD323	Integrin b7
CD1c	CD80	CD357	XCR1
CD200	CD81	CD36	Podoplanin
CD200R	CD82	CD369	Rat IgG2b ctrls
CD202b	CD83	CD370	CD132
CD203c	CD85g	CD371	CD195
CD205	CD85k	CD45RO	CX3CR1
CD206	CD87	CD51	Rat IgM ctrl
CD207	CD89	CD59	SSEA-3
CD21	CD8a	CD7	
CD213a1	CD9	CD71	
CD213a2	CD90	CD84	
CD218a	CD93	CD88	
CD221	CD94	CRTAM	
CD223	CD95	HER-3	
CD226	CD96	FPR3	
CD227	CD97	Ganglioside GD2	
CD229	CD99	GPR83	
CD23	CXCL16	HLA-ABC	
CD231			

Figure S2: Antigens screened by the Human Cell Surface Marker Screening Kit from Biolegend® (LEGENDscreen). Each plaque was detailed in one column.

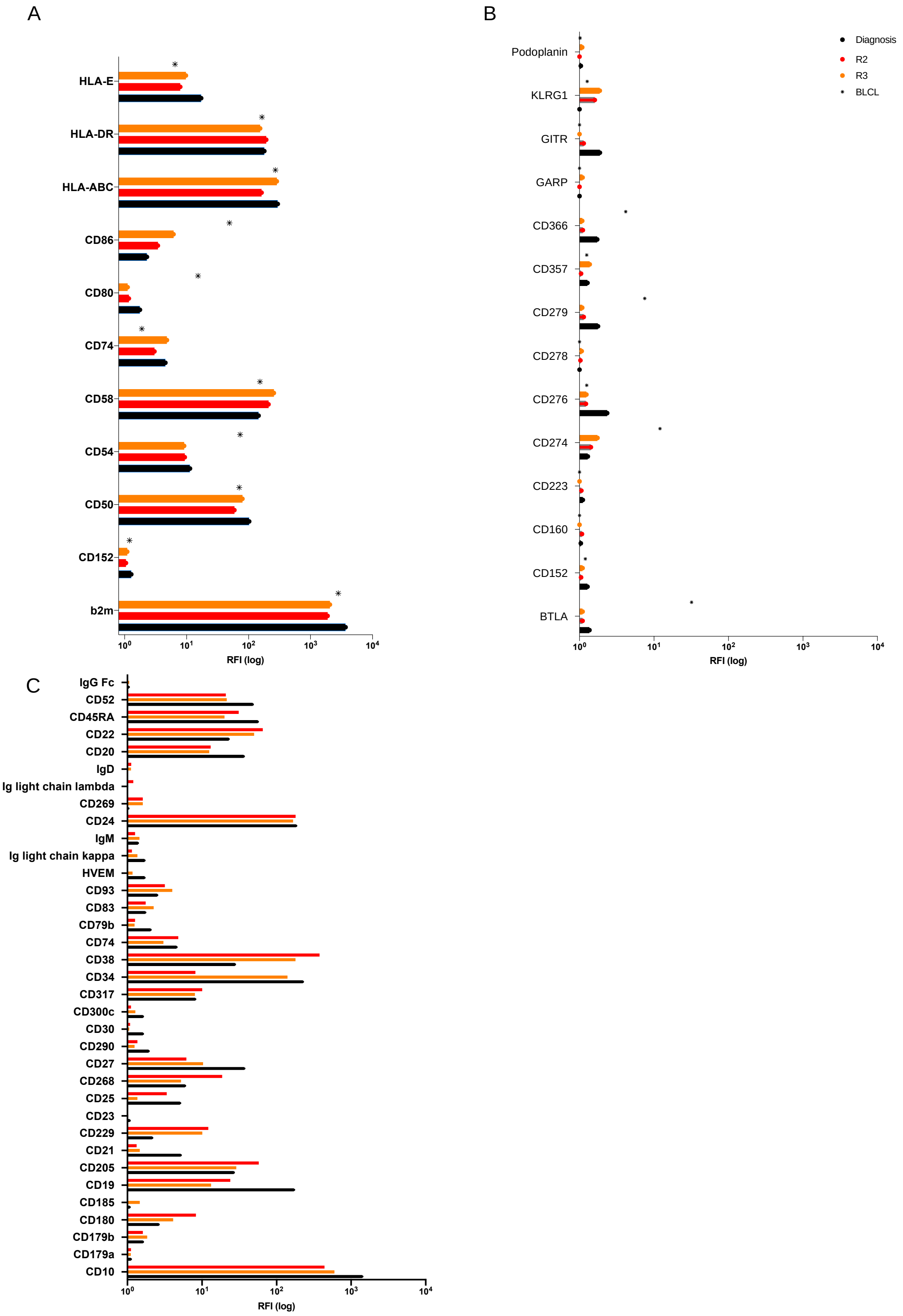

Figure S3: Immunophenotyping results: Immunophenotyping results for the 3 ALL: at diagnosis (Dg), post-HSCT relapse (R2) and post-CAR-T relapse (R3). A: molecules involved in the immunological synapse; B: checkpoint inhibitors and C: B-cells markers. The immunophenotype of a B-EBV induced cell line (BLCL) was presented as a control.

CD205; CD200R; CD183; CD18; CD179a; CD172αβ; CD170; CD16; CD156c; CD15; CD14; CD137L; CD13; CD11b; CD11a; CD107a; β2microglobulin; CCR10; CD97; CD95; CD93; CD89; CD82; CD74; CD64; CD63; CD58; CD55; CD50; CD48; CD47; CD45; CD44; CD43; CD367; CD354; CD35; CD33; CD31; CD298; CD277; CD27; CD263; Class I HLA; GPR83; GD2; FPR3; CD88; CD59; CD45RO; CD371; CD369; CD301; CD284; CD282; CD269; CD243; CD24; CD217; CD199; CD197; CD184; CD158b; CCR8; TM4SF20; TCRαβ; Siglec-8; MSC (W7C6); IL-21 R; CD195; CD132; CD85d; CD85a; CD49f; CD294; CD210; CD120b; CD201; CD115; CD66b; Sialyl Lewis X; CD92; CD66ace; CD368; CD32; CD274; CD181; CD11c; MSC (W3D5); LOX-1s; Integrinβ5.

Figure S4: Cell surface antigen expressed by the monocytic population isolated from the R3 sample.
